# Foveal Hypoplasia Grading with Optical Coherence Tomography: Agreement and Challenges Across Experience Levels

**DOI:** 10.3390/diagnostics15060763

**Published:** 2025-03-18

**Authors:** Riddhi Shenoy, Gail D. E. Maconachie, Swati Parida, Zhanhan Tu, Abdullah Aamir, Chung S. Chean, Ayesha Roked, Michael Taylor, George Garratt, Sohaib Rufai, Basu Dawar, Steven Isherwood, Ryan Ramoutar, Alex Stubbing-Moore, Esha Prakash, Kishan Lakhani, Ethan Maltyn, Jennifer Kwan, Ian DeSilva, Helen J. Kuht, Irene Gottlob, Mervyn G. Thomas

**Affiliations:** 1 The University of Leicester Ulverscroft Eye Unit, Robert Kilpatrick Clinical Sciences Building, School of Psychology and Vision Sciences, Leicester LE2 7LX, UK; sr670@leicester.ac.uk (R.S.); g.d.maconachie@sheffield.ac.uk (G.D.E.M.); zt33@leicester.ac.uk (Z.T.); abdullah.aamir@roche.com (A.A.); ar618@student.le.ac.uk (A.R.); george.garratt@nhs.net (G.G.); sohaib.rufai@leicester.ac.uk (S.R.); kishanlakhani@doctors.org.uk (K.L.); ethan.maltyn@uhl-tr.nhs.uk (E.M.); helen.bauckham1@nhs.net (H.J.K.); ig15@le.ac.uk (I.G.); 2 Division of Ophthalmology and Orthoptics, Health Science School, University of Sheffield, Sheffield S10 2TN, UK; 3 Department of Ophthalmology, University Hospitals of Leicester, Leicester Royal Infirmary, Leicester LE1 5WW, UK; swati.parida@uhl-tr.nhs.uk (S.P.); chung.s.chean@uhl-tr.nhs.uk (C.S.C.); basu.dawar@uhl-tr.nhs.uk (B.D.); stevenisher@gmail.com (S.I.); ryan.ramoutar@uhl-tr.nhs.uk (R.R.); alexander.moore11@nhs.net (A.S.-M.); jennifer.kwan27@gmail.com (J.K.); ian.desilva@uhl-tr.nhs.uk (I.D.); 4 Department of Ophthalmology, Nottingham University Hospitals, Nottingham NG7 2UH, UK; 5 Cooper Neurological Institute and Cooper Medical School of Rowan University, Camden, NJ 08002, USA

**Keywords:** fovea, albinism, paediatric ophthalmology, optical coherence tomography

## Abstract

**Background/Objectives**: The diagnosis and prognosis of arrested foveal development or foveal hypoplasia (FH) can be made using the Leicester grading system for FH and optical coherence tomography (OCT). In clinical practice, ophthalmologists and ophthalmic health professionals with varying experience consult patients with FH; however, to date, the FH grading system has only been validated amongst experts. We compare the inter-grader and intra-grade agreement of healthcare professionals against expert consensus across all grades of FH. **Methods**: Handheld and table-mounted OCT images (*n* = 341) were graded independently at a single centre by experts (*n* = 3) with over six years of experience and “novice” medical and allied health professionals (*n* = 5) with less than three years of experience. Sensitivity, specificity, and Cohen’s kappa scores were calculated for each grader, and expert vs. novice performance was compared. **Results**: All graders showed high sensitivity (median 97% (IQR: 94–99)) and specificity (median 94% (IQR: 90–95)) in identifying the presence or absence of FH. No significant difference was seen in specificity between expert and novice graders, but experts had significantly greater diagnostic sensitivity (median difference = 5.3%, H = 5.00, *p* = 0.025). Expert graders had the highest agreement with the ground truth and novice graders showed great variability in grading uncommon grades, such as atypical FH. The proposed causes of misclassification included macular decentring in handheld OCT scans in children. **Conclusions**: Ophthalmologists of varying experience and allied health professionals can accurately identify FH using handheld and table-mounted OCT images. FH identification and paediatric OCT interpretation can be improved in wider ophthalmic clinical settings through the education of ophthalmic staff.

## 1. Introduction

Foveal development processes start in early gestation and continue for several years after birth [1]. Foveal hypoplasia (FH) is characterised by the continuation of the inner retinal layers posterior to the foveola and is caused by the disruption of the foveal development process at any stage [1]. This can occur in conditions such as albinism, *PAX6* mutations, *SLC38A8* mutations, retinopathy of prematurity, and optic nerve hypoplasia [1,2]. Non-invasive optical coherence tomography (OCT) can be used to assess foveal maturity including the formation of a foveal pit, the extrusion of the inner retinal layers, a thickened outer nuclear layer, and long outer segments [3].

The classification of FH using OCT is clinically valuable as it can diagnose and predict visual prognosis [3]. The Leicester grading system for the structural grading of OCT morphology differentiates between typical and atypical FH, which affects the outer retinal layers and occurs in conditions such as achromatopsia [3,4,5,6]. Higher grades of typical FH, representing more abnormal foveal development, are significantly associated with poorer visual outcomes in those with albinism, nystagmus, and a number of associated conditions [2,3,7,8,9,10]. FH grading has also been shown to be the strongest predictor of visual acuity in albinism and nystagmus, compared to ocular hypopigmentation and other methods of assessing visual acuity in children [7,9].

Recent findings from the Foveal Development Investigators Group have also highlighted that FH grading can provide important diagnostic clues for about underlying genotypes. For instance, *SLC38A8* mutations are exclusively associated with high grades of FH (grade 3 or 4), and syndromic forms of oculocutaneous albinism similarly present only with higher grades of FH [2,11]. These genotype–phenotype correlations reinforce the value of structured OCT-based classification, not only for clinical assessment but also for refining diagnostic pathways in inherited retinal disorders [2].

The OCT grading of FH provides a practical and widely applicable method for assessing foveal development, offering a standardised approach to predicting visual outcomes and guiding clinical management. However, parameters measured from OCT alone, such as total foveal thickness and pit depth, do not reliably predict best-corrected visual acuity (BCVA), particularly in conditions such as albinism [1]. Photoreceptor morphology, including the length of the cone outer segment, has been shown to correlate more strongly with both cone density and visual function [12]. However, these high-resolution metrics require specialised imaging techniques, such as adaptive optics, which are not routinely available in clinical practice.

Given the wide spectrum of phenotypes amongst people with FH, accurate OCT grading is necessary for the accessible and accurate prediction of future vision in clinical settings [2,3]. This has particular significance for parents of affected children who may have anxiety or fear about how their child’s vision could impact their future [13]. Understanding the level of vision in a preverbal child can help parents plan adjustments for children to support their development and educational attainment [7].

In clinical practice, FH and associated conditions may present to a range of ophthalmology clinical professionals with varying experience or knowledge of FH and the significance of its grading. Validation of the grading system has primarily used expert human graders, and an artificial intelligence model has even been shown to reliably estimate FH grades [14]. While accurate grading has wide clinical applicability, the level of experience required to grade FH using OCT data is unclear. This study is the first to measure the accuracy of FH grading between novice and expert human graders.

## 2. Materials and Methods

### 2.1. Clinician Grader Profile

The graders consisted of eight individuals recruited from the University Hospitals Leicester NHS trust and East Midlands deanery, with a range of less than 1 year to 10 years of clinical experience in interpreting paediatric OCT images. The expert (*n* = 3) and novice (*n* = 5) graders consisted of both medical professionals and allied health professionals (Table 1). This included three male and five female clinicians. All clinicians were blinded to the diagnoses and patient records.

### 2.2. Leicester Grading System for Foveal Hypoplasia [3]

**Figure 1:**
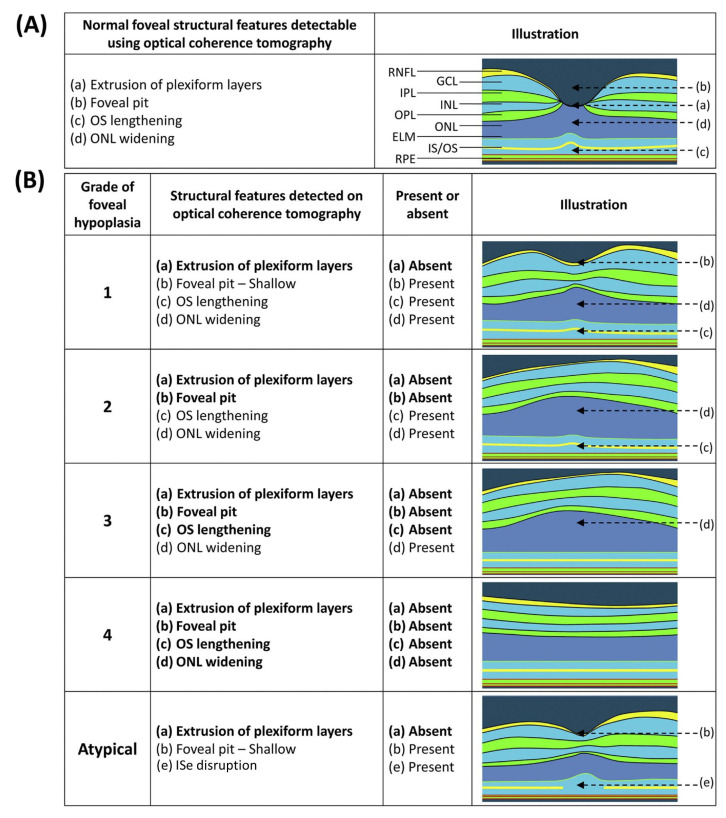
(**A**) Illustration showing the unique features of a normal fovea detectable on optical coherence tomography. (**B**) Illustration of typical and atypical grades of FH. All grades of FH had incursion of the inner retinal layers. Atypical FH also had incursion of the inner retinal layers. Grade 1 FH is associated with a shallow foveal pit, outer nuclear layer (ONL) widening, and outer segment (OS) lengthening relative to the parafoveal ONL and OS length, respectively. In Grade 2 FH, all features of grade 1 are present except the presence of a foveal pit. Grade 3 FH consists of all features of grade 2 FH except the widening of the cone outer segment. Grade 4 FH contains all the features seen in grade 3 except there is no widening of the ONL at the fovea. Finally, an atypical form of FH also is described in which there is a shallower pit with a disruption of the inner segment ellipsoid (ISe), possibly a sign of photoreceptor degeneration. ELM = external limiting membrane; GCL = ganglion cell layer; INL = inner nuclear layer; IPL = inner plexiform layer; OPL = outer plexiform layer; RNFL = retinal nerve fibre layer; RPE = retinal pigment epithelium (Adapted from [3].

The Leicester grading system describes grades 1–4 of typical FH and one grade of atypical FH. Notably, all grades of FH, including atypical FH, feature incursion of the inner retinal layers. Atypical FH is differentiated by the disruption of the junction of the inner segment and outer segment of the photoreceptor. The grades of typical FH are determined by the identification of features such as outer segment lengthening, the presence of the foveal pit, and outer nuclear layer widening (Figure 1).

Grade 1 FH is associated with the presence of a foveal pit, outer nuclear layer (ONL) widening, and outer segment (OS) lengthening relative to the parafoveal ONL and OS length, respectively. In Grade 2 FH, all features of grade 1 are present except there is no foveal pit. Grade 3 FH consists of all features of grade 2 FH except the widening of the cone outer segment. Grade 4 FH contains all the features seen in grade 3 except there is no widening of the ONL at the fovea [3].

### 2.3. Dataset

A total of 341 OCT scans were obtained retrospectively at the University of Leicester from two different OCT machines, one handheld and one table-mounted. OCT image acquisition has already been described in previous publications [6,15]. The ground truth data were obtained from the patients’ records and then further reviewed and graded by an expert consensus panel.

The dataset included a range of FH grades and comprised 133 OCT scans without FH, 106 scans with grade 1 FH, 43 with grade 2 FH, 42 with grade 3 FH, 11 with grade 4 FH, and 6 scans with atypical FH.

### 2.4. Statistical Analysis

To evaluate the accuracy and agreement of FH grading among graders of varying experience levels, several statistical methods were employed. These methods included sensitivity and specificity calculations, inter-grader agreement assessments, and statistical tests to compare the performance of novice and expert graders. The sensitivity and specificity of each grader’s ability to correctly identify the presence or absence of FH were calculated. Sensitivity was defined as the proportion of true positive cases (correct identifications of FH) detected out of all FH cases, while specificity was defined as the proportion of true negative cases (correct identifications of normal foveal morphology) detected out of all normal cases. To evaluate how closely each grader’s classifications aligned with the expert consensus (the ground truth), Cohen’s kappa scores were calculated for each grader. In addition to comparing individual graders against the ground truth, inter-grader agreement was assessed to evaluate consistency across graders. Pairwise Cohen’s kappa was calculated between all grader pairs to measure the level of agreement between them, which helped identify patterns of consistency or variability in grading. The performance of novice graders (with less than 5 years of experience) was compared to that of expert graders (with more than 5 years of experience) using the Mann–Whitney U test given the non-parametric nature of the data.

## 3. Results

### 3.1. Sensitivity and Specificity

**Figure 2:**
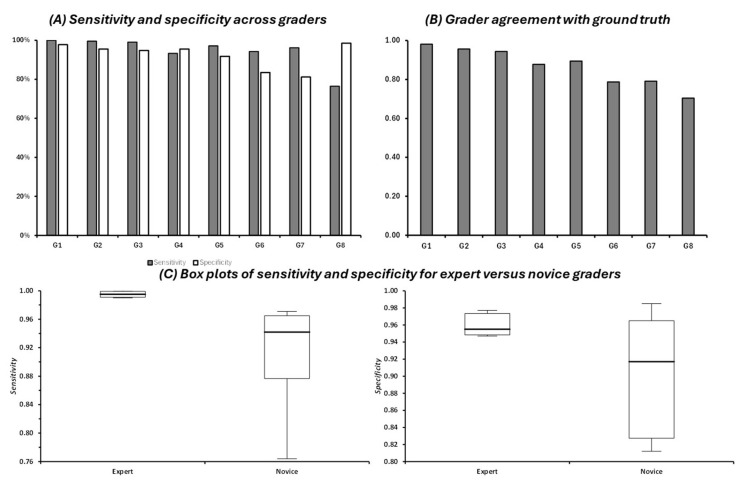
(**A**) Distribution of sensitivity and specificity of identifying FH by grader. (**B**) Inter-grader agreement assessed by Cohen’s kappa scores, comparing each grader’s classification to the ground truth. (**C**) Box plots of sensitivity and specificity for expert versus novice graders.

Overall, graders were able to accurately detect FH if it was present, with a median sensitivity of 97% (IQR: 94–99). All graders were also able to accurately exclude FH if it was absent, essentially meaning they were able to identify a normal macular OCT, with a median specificity of 94% (IQR: 90–95) (Figure 2). The expert graders (G1–G3) exhibited the highest sensitivity (100–99.04%) and specificity (97.74–94.74%). The novice graders (G4–G8) demonstrated a range of sensitivities (97.12–76.44%) and specificities (98.50–81.20%) (Figure 2A). There was a significant difference in diagnostic sensitivity between expert and novice graders (median difference = 5.3%, H = 5.00, *p* = 0.025, Figure 2C). However, no significant difference was seen in specificity between expert and novice graders (median difference = 3.8%, H = 0.81, *p* = 0.37, Figure 2C).

### 3.2. Inter-Grader Agreement

To evaluate the accuracy of each grader in comparison to the ground truth, Cohen’s kappa scores were calculated using each grader’s classification and the ground truth (Figure 2B). Expert graders (G1–G3) demonstrated the highest agreement (0.98–0.94) with the ground truth. Graders 4 and 5 exhibited substantial agreement with kappa scores of 0.88 and 0.89. Graders 6, 7, and 8, with the least experience, had the lowest kappa scores (0.70–0.79).

**Figure 3:**
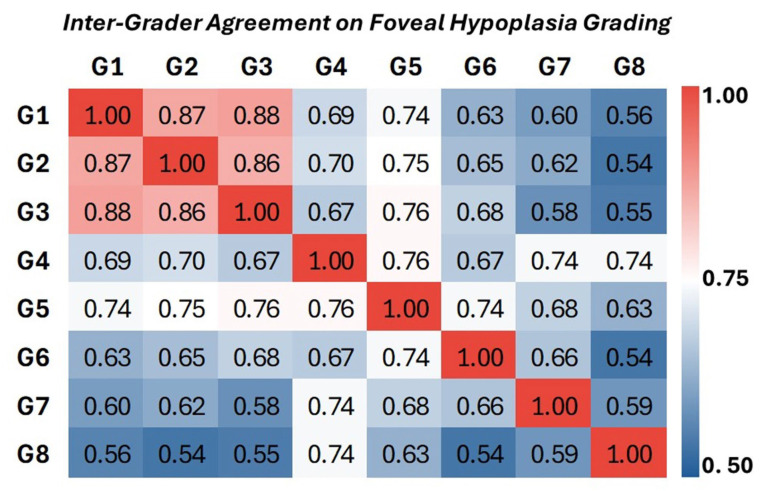
Pairwise inter-grader agreement for FH grading, assessed using Cohen’s kappa. The heatmap illustrates the level of agreement between each pair of graders, with kappa values ranging from 0.54 to 1.0. Lower kappa values (blue) indicate less agreement, while higher values (red) indicate greater agreement. The midpoint (white) represents moderate agreement.

We next compared the consistency of FH grading among different clinicians using pairwise Cohen’s kappa scores for all possible pairs of graders. This analysis revealed the level of agreement between each pair of graders, providing insight into the variability in grading across individuals with different levels of experience. The results are shown in the heatmap in Figure 3. The highest pairwise kappa scores were observed between Graders 1, 2, and 3, all of whom were considered experts. The kappa values between these graders ranged from 0.85 to 0.89. Among less experienced graders (G7–G8) the pairwise kappa ranged from 0.74 to 0.54, indicating more variability and less consistency in grading and a greater tendency to deviate from the consensus (Figure 3).

### 3.3. Breakdown Across Grades

**Figure 4:**
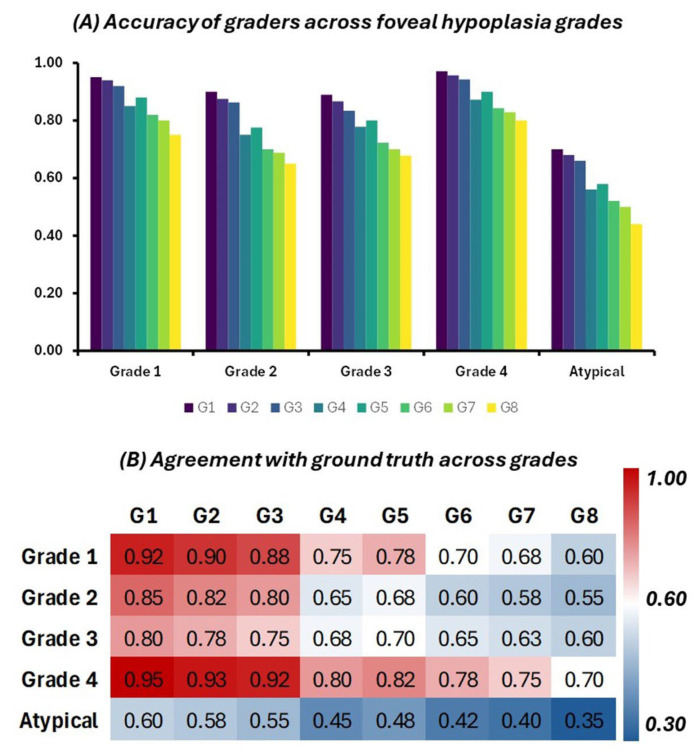
(**A**) Accuracy of each grader in identifying FH across different grades. Higher bars indicate greater accuracy in classification relative to the ground truth. (**B**) Heatmap of Cohen’s kappa scores, representing the agreement between each grader’s classification and the ground truth across different grades of FH. Warmer colours represent higher agreement, while cooler colours represent lower agreement.

The accuracy of each grader in identifying FH was assessed across different grades (grade 1, grade 2, grade 3, grade 4, and atypical). The grouped bar chart (Figure 4A) illustrates these findings, showing how accurately each grader classified the scans in comparison to the ground truth. Expert graders (G1 to G3) demonstrated consistently high accuracy across all grades, with particularly strong performances in grades 1 and 4. For instance, Grader 1 (G1) achieved an accuracy of 95% for grade 1 and 97% for grade 4. In contrast, novice graders (G4 to G8) showed greater variability in their performance, with lower accuracy observed particularly in more complex or less common grades, such as atypical FH. Grader 8 (G8), for example, exhibited the lowest accuracy of 44% for the atypical grade, indicating the challenges faced by less experienced graders in identifying more subtle or atypical presentations.

To further assess the agreement between each grader’s classification and the ground truth, Cohen’s kappa scores were calculated for each grader across all grades. These results are presented in a heatmap (Figure 4B), where colour intensities reflect the level of agreement. The heatmap indicates that expert graders (G1 to G3) consistently achieved high kappa scores across all grades, with kappa values close to 0.95 in grades 1 and 4, reflecting almost perfect agreement with the ground truth. The kappa scores for novice graders (G4 to G8) were generally lower, particularly in more complex grades such as atypical FH. Grader 8 (G8), for instance, had the lowest kappa score of 0.35 for the atypical grade, indicating a relatively low level of agreement with the ground truth in this category.

### 3.4. Misclassified Cases

**Figure 5:**
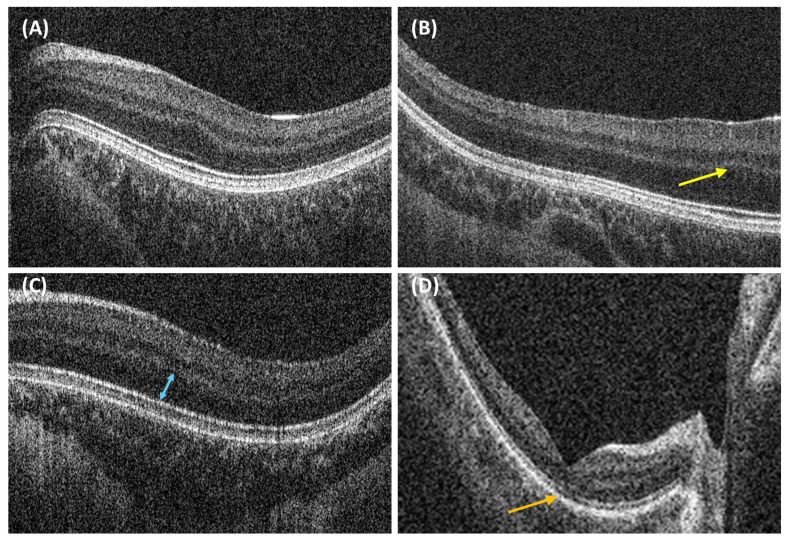
Examples of misclassified cases of FH. (**A**) Grade 2 expert consensus with subtle OS lengthening and absence of foveal pit. (**B**) Grade 2 expert consensus, with fovea not centred on handheld OCT image; see fovea (yellow arrow). (**C**) Grade 3 expert consensus due to subtle ONL widening (blue arrow) and absent OS lengthening. (**D**) Atypical expert consensus; note disruption of inner segment ellipsoid (ISe) (orange arrow).

Grade 2 and atypical FH were most frequently misclassified, and more so amongst novice graders. A few examples of misclassification are presented in Figure 5. On review, images of grade 2 cases misclassified as higher grades of FH had more subtle OS lengthening (Figure 5A). Images of grade 3 cases misclassified as grade 4 FH had more subtle ONL widening (Figure 5C).

While the prevalence of atypical cases of FH was low in this dataset, these were frequently misclassified as normal by novice graders. This highlights the need for clinicians to be aware that changes in the inner segment ellipsoid may exist despite the observation of the features of normal foveal structures, such as the presence of a foveal pit (Figure 5D).

### 3.5. Implications for Paediatric OCT

Notably, one image (Figure 5B) of grade 2 FH was misclassified by seven graders as grade 4, which was deemed likely to be due to the macula being off-centre. This highlights the need for clinicians grading handheld OCT images in children to be aware that these may be difficult to acquire and, due to fixation instability and nystagmus, the macula may not always be centred in these images.

## 4. Discussion

In this study, ophthalmologist and orthoptist graders of all levels of experience were able to accurately identify OCT scans with normal foveal development with a median specificity of 94% (IQR: 90–95). This high specificity aligns with expectations, given the graders’ prior clinical experience in ophthalmology. Graders of all levels of experience were also able to use the Leicester grading system to accurately identify the presence of FH, with a median sensitivity of 97% (IQR: 94–99). However, expert graders had significantly greater diagnostic sensitivity compared to novice graders, particularly for atypical FH.

Expert graders have been found to have greater diagnostic sensitivity compared to novice graders in similar studies [16,17]. One study observed inter-reader variability in grading diabetic retinopathy in 400 fundus images using the Early Treatment Diabetic Retinopathy Study (ETDRS)’s standard photographs [17]. Amongst 12 readers from different professional backgrounds, the sensitivity of identifying referrable diabetic retinopathy ranged between 0.7 and 1.0, with retinal specialists having the highest specificity (0.95–0.97). The same study highlighted the value of professionals such as ophthalmic photographers and ophthalmic nurses in accurately identifying referrable retinopathy, and the inter-observer agreement for identifying retinopathy severity and maculopathy was similar in these groups compared to in general ophthalmologists [17].

Grade 1 and 4 of FH generally had the highest agreement with the ground truth across all graders in this study, with lower agreement seen for grades 2 and 3 and atypical FH. Our study reported higher rates of agreement in qualitative grading compared to similar studies using the Frisén classification for disc swelling [18,19]. These studies report a wide range of inter-grader agreement, from 1.6% to 48%, in fundus images of patients with idiopathic intracranial hypertension [18,19]. It was suggested that the lack of agreement may be due to this classification not accounting for other disc changes, such as haemorrhages, leaving room for subjective interpretation [19]. Similarly, in our study, FH grades did not account for other macular pathologies, which could be a barrier to accurate grading, particularly for less experienced graders.

Atypical grades were the most frequently misclassified but comprised the smallest group in the dataset (6 cases), reducing the reliability of the inter-grader agreement found in this study. Atypical FH may be easier to miss, particularly for inexperienced graders, given that it may be present despite other features of normal foveal morphology. Achromatopsia is characterised by progressive ONL thinning, ellipsoid zone disruption, and an increased foveal hyporeflective zone, which may be more marked towards the nasal side [6]. Achromatopsia has been shown to slow retinal development and it has been suggested that emerging gene therapies could preserve subsequent retinal development if achromatopsia is identified and diagnosed early [20].

Expert graders consistently demonstrated higher accuracy and agreement across all grades compared to novice graders. For example, the accuracy of expert graders in identifying grade 1 FH was approximately 95%, compared to the 75% among novice graders. Similarly, Cohen’s kappa scores were significantly higher among experts, indicating their almost perfect agreement with the ground truth, particularly in grades 1 and 4. This underscores the critical role of experience in interpreting OCT images and suggests that targeted training for novice graders could help mitigate the observed variability in grading accuracy.

Our study highlights a need for education on handheld OCT and the grading of FH amongst clinical ophthalmology professionals. With greater ease of use and portability compared to table-mounted OCT, handheld OCT has been proposed to be highly accessible for non-specialists to use in a range of patients and settings [21]. In particular, while standard table-mounted OCT devices may be used in cooperative children as young as 3 years of age, handheld OCT is feasible in infants from birth, without sedation, in the clinic setting [22]. This study highlighted a need for better familiarity with handheld OCT images, particularly surrounding the identification of the fovea, compared to table-mounted OCT. Factors found to facilitate the successful acquisition of handheld OCT images of the optic nerve in children include the use of an assistant to help with maintaining engagement with visual fixation devices [22]. Beyond OCT imaging, fundus handheld imaging is becoming more popular due to its portability and the possibility of enabling telemedicine. Previous studies looking at handheld fundus imaging comparing image acquisition, gradeability, and the patient experience of different handheld instruments reported the best overall performance from two handheld fundus cameras: the Remidio Non-Mydriatic Fundus On Phone (NMFOP) (Bengaluru, India), an infrared smartphone-based fundus camera, and the Volk Pictor Plus (Mentor, OH, USA), a non-mydriatic fundus camera with posterior (retinal) and anterior imaging modules [23].

There is a wide variety of evidence of medical education for image interpretation, with one review of 81 papers describing the optimal modes for learning using theories within cognitive psychology, such as diagnostic reasoning [24]. The authors of the review concluded that a balance of both non-analytic reasoning, or ‘pattern recognition’, and analytic reasoning, the careful identification of all features in an image to improve diagnostic accuracy, was necessary [24]. This theory could be interpreted in a manner that supports the use of different modes of image-interpreting education. For example, a didactic lecture or an online module to help clinicians understand and identify OCT features in FH is a mode for teaching analytic reasoning, while an online reference guide of OCT images of varying grades or an online case-based practice module to aid pattern recognition are methods for teaching non-analytic reasoning. Future work could test such interventions to find the most appropriate intervention to improve the recognition and grading of FH amongst different professional groups. Some studies have highlighted the value of eye tracking in gauging the accuracy of the interpretation of images amongst professionals of different levels [25].

The limitations of this study included that graders were only recruited from a single centre with a strong track record using handheld OCT both clinically and within research. This may reduce the generalisability of these findings. Future work could involve recruiting graders from multiple sites to strengthen their external validity. Furthermore, there was a relatively small subset of atypical FH cases (*n* = 6), which also reduces reliability of the findings in this group. This could be improved in future work by using imaging datasets with more balanced subgroups of FH grades.

Considering the diagnostic and prognostic value of accurately grading foveal hypoplasia, a deep learning approach could provide more a consistent and large-scale identification of FH. Indeed, bespoke artificial intelligence models have been shown to accurately recognise and grade FH [14,26]. In addition, educational resources could be developed to support the incorporation of grading FH and the use of handheld OCT into the ophthalmology training curricula. These resources could also be used in the training of allied health professionals such as ophthalmic nurses, orthoptists, and ophthalmic imaging technicians to support the grading ability and upskilling of these professional groups to ultimately improve patient outcomes.

## Figures and Tables

**Table 1:** Clinician grader profile showing years of experience in interpreting OCT images and professional qualification of each grader.

Grader	Grading Experience (Years)	Professional Role
1	10	Ophthalmologist
2	10	Orthoptist
3	6	Ophthalmologist
4	3	Orthoptist
5	2	Ophthalmologist
6	1	Ophthalmologist
7	<1	Postgraduate doctor
8	<1	Ophthalmologist

## Data Availability

Dataset available on request from the authors.

## Abbreviations

The following abbreviations are used in this manuscript:

FH	Foveal hypoplasia
OCT	Optical coherence tomography
ONL	Outer nuclear layer
OS	Outer segment
